# T cell dysregulation in inflammatory diseases in ICU

**DOI:** 10.1186/s40635-022-00471-6

**Published:** 2022-10-24

**Authors:** Marta Luperto, Lara Zafrani

**Affiliations:** 1 Human Immunology and Immunopathology, INSERM U 976, University Paris Cite, 75010 Paris, France; 2 Medical Intensive Care , Unit, Assistance Publique Des Hôpitaux de Paris, Saint Louis Hospital, 1, Avenue Claude Vellefaux, 75010 Paris, France

## Abstract

Severe inflammatory diseases, including sepsis, are characterized by an impaired host adaptive and innate immunity which results in immunosuppression, responsible for secondary infections and increased morbidity and mortality in critically ill patients. T cells are major actors of the immune system. During post-aggressive immunosuppression, lymphopenia, reduction of innate T cells, changes in T helper cell polarization and regulatory T cell increase are observed. The main mechanisms involved in T cell dysregulation are T cell apoptosis, autophagy deficiency, T cell anergy, T cell exhaustion and T cell metabolic reprogramming. In this review, we describe the alterations of T cell regulation, their mechanisms, and their association with clinical outcomes in severe inflammatory diseases, foremost of which is the sepsis.

## Take home message

This review focuses on the alterations of T cell regulation and their mechanisms in severe inflammatory ICU diseases. Lymphopenia, reduction of innate T cells, changes in T helper cell polarization and regulatory T cell increase contribute to secondary immunosuppression in ICU patients.

## Introduction

Pro-inflammatory host response mediated by the activation of the innate immune system is a hallmark of many critical diseases of infectious (sepsis) or non-infectious causes, such as trauma, pancreatitis, or burns [1]. Innate immunity plays a crucial role in the activation of later antigen-specific adaptive immunity. However, in parallel with the inflammatory phase, dysfunction of the immune system may occur, that is called post-aggressive immunosuppression or compensatory anti-inflammatory response syndrome. This dysfunction involves both the adaptive and the innate immune system [2, 3].

Lymphopenia is a hallmark of this immunosuppression and is often present at Intensive Care Unit (ICU) admission. Its persistence after the 3rd day of ICU admission is strongly associated with secondary infections and mortality [4, 5]. Indeed, patients admitted in ICU for a non-septic disease with a loss of lymphocytes for more than 3 days have a greater risk of nosocomial sepsis [6]. Moreover, sustained immunosuppression predisposes septic patients to secondary opportunistic infections, including fungal infections [7] or latent virus reactivations [8–10].

Holhstein et al. have shown that decrease of lymphocyte count can be observed at ICU admission without any difference between septic or non-septic patients. ICU survivors showed dynamic changes towards restoration of lymphopenia and T cell depletion, while non-survivors failed to restore lymphocyte counts [11].

**Fig. 1:**
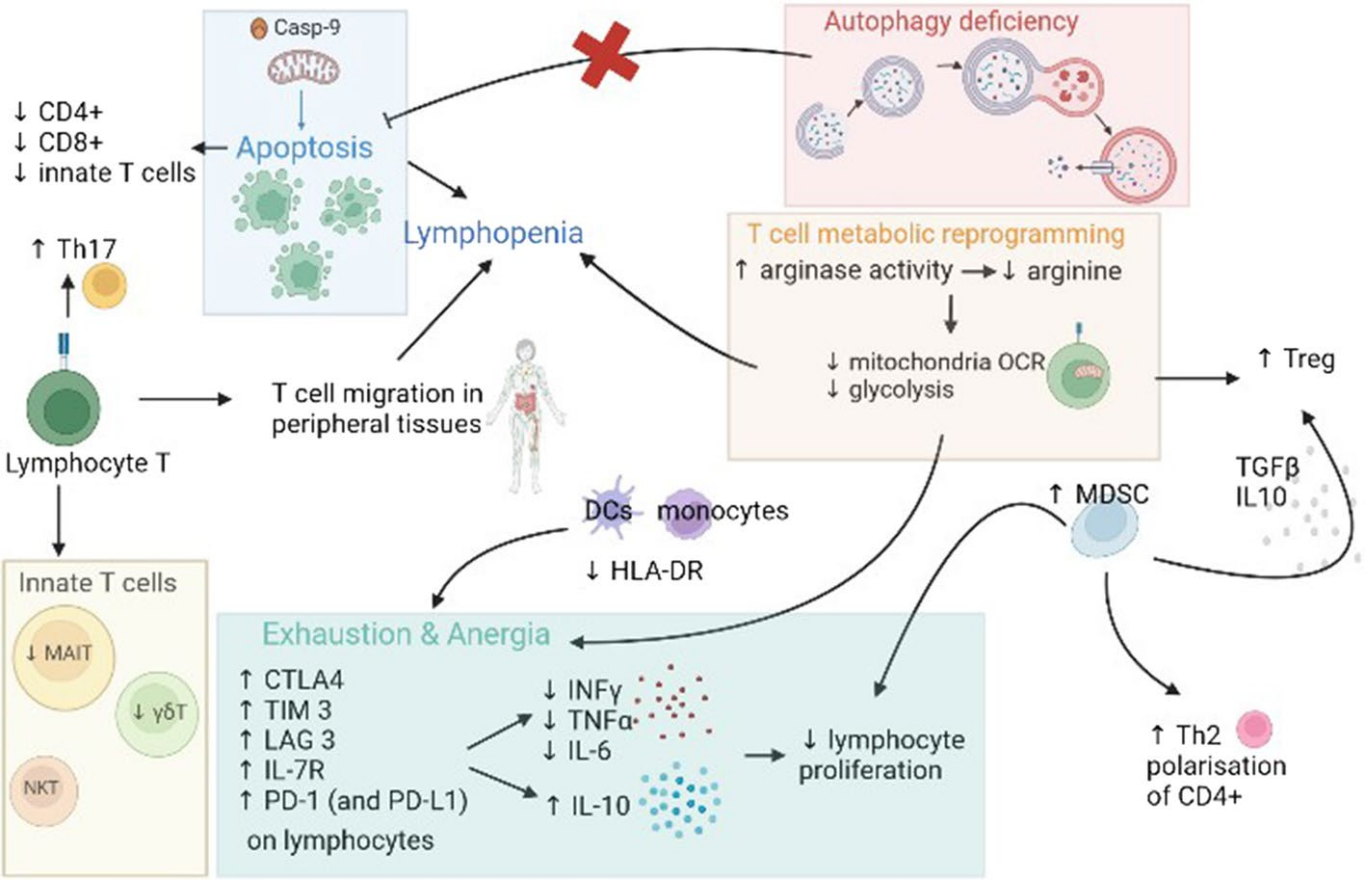
T cells dysregulation in critical illnesses. T cell alterations include lymphopenia with polarization of Th cells towards Th2, increase of Th17 cells, increase of Tregs and reduction of innate T cells (MAIT cells and γδT cells). The main mechanisms responsible for these alterations are: lymphocyte apoptosis, autophagy deficiency, lymphocyte metabolic reprogramming and T cell anergy and exhaustion. Lymphocyte apoptosis: in the mitochondrial pathway, caspase-9 serves as an initiator caspase. The activation of the initiator caspase, caspase-9 (mitochondrial pathway) is responsible for increased apoptosis. Autophagy deficiency results in reduced lymphocyte apoptosis inhibition. Lymphocyte metabolic reprogramming: during sepsis or burns, the increase of arginase activity induces a decrease of arginine with a subsequent reduction of mitochondrial activity and glycolysis. Exhaustion and anergy: in critical inflammatory diseases, T cells express exhaustion markers on their surface and have reduced capacity to proliferate and activate. They produce less pro-inflammatory cytokines and more anti-inflammatory cytokines, such as IL-10. Finally, alterations of innate immune response contribute to T lymphocytes dysfunction. Monocytes and dendritic cells express less HLA-DR and have therefore, reduced capacities of antigen presentation, thus contributing to T cells anergy and exhaustion. NK cells produce less INFγ. The increase in MDSC is involved in the Treg increase, as well as in the Th2 cells polarization, in the inhibition of T cell proliferation and in the inhibition of NK cells cytokines production. ATP: adenosine triphosphate; Th2: T helper 2 cells; Th17: Th17 cells, Casp-9: caspase 9, OCR: oxygen consumption rate, DC: dendritic cells, HLA-DR: human leukocyte antigen-DR isotype, CTLA-4: cytotoxic T-lymphocyte-associated protein 4, TIM-3: T-cell immunoglobulin and mucin containing protein-3, LAG-3: lymphocyte-activation gene 3, PD-1: programmed death protein 1, INFγ: interferon γ, TNFα: tumor necrosis factor α, IL-6: interleukin 6, IL-10: interleukin 10, TGF-β: transforming growth factor β, Th2: Th2 cells, Th17: Th17 cells, MDSC: myeloid-derived suppressor cells

Post-aggressive immunosuppression may affect different subsets of T cells, including conventional and non-conventional (or innate) T cells. In this review, we will focus on the main dysfunctions of conventional and innate T cells in critical inflammatory diseases, foremost of which is the sepsis (Fig. 1).

## T lymphocytes

### T lymphocytes dysregulation in critical diseases

#### Alteration of circulating T lymphocytes

During sepsis, both CD (cluster of differentiation) 4 + and CD8 + T lymphocytes are reduced in the early phase of the disease. Montserrat et al*.* have demonstrated different kinetic patterns of T cell subsets in septic patients. Whereas restoration of CD4 + cells count was usually observed by day 14 in survivors, the decrease of CD8 + level persisted over a longer period of time [12]. Moreover, there was a reduction of the non-effector subset, CD45RA + CD45RO- for both CD4 + and CD8 + that remained low even after sepsis resolution. This subset was lower in survivors when compared with non-survivors. Effector subsets (CD45RA + CD45RO +) were reduced during the first week of ICU admission in septic patients (versus healthy donors) and in survivors (versus non-survivors). After 7 days, effector subsets recovered in survivors. Finally, authors analyzed the expression of CD28 (a costimulatory molecule that is lost when the lymphocyte is activated) and CD62L antigen (a homing receptor responsible for migration of circulating T lymphocytes to peripheral lymph nodes) on CD4 + and CD8 + T lymphocytes. They showed that lower numbers of circulating CD8 + CD28 + , CD4 + CD28 + and CD8 + CD62L + , CD4 + CD62L + were predictive of a better outcome. Taken together, their data suggest time differences in the recirculation of T lymphocytes between survivors and non-survivors. The authors hypothesized that a slower migration of naïve and effector cells contribute to delayed tissue response and worse outcome [12].

Similarly, CD4 + and CD8 + T lymphocytes have been shown to be reduced and functionally impaired during SARS-CoV-2 (severe acute respiratory syndrome-coronavirus 2) infection [13]. These changes have been associated with the severity of the disease and the risk for ICU admission [14]. T cell redistribution and sequestration into infected organs (especially the lungs) may also contribute to lymphopenia in critically ill COVID-19 patients [14].

#### *T CD4* + *cell polarization*

During sepsis, among the CD4 + cells, a shift from a Th (T helper)1 to a Th2 cell profile may contribute to sepsis-induced immunosuppression [15]. Indeed, Ferguson et al*.* compared Th1 and Th2 subsets in septic patients with non-septic critically ill patients and found that Th2 immune response was predominant in septic patients [6]. Watanabe et al*.* found that mice that were predisposed to the Th2 response had increased mortality during sepsis when compared to mice predisposed to the Th1 response [16]. Conversely, there is an increased proportion of Th17 CD4^+^ lymphocytes, and Th17 count is higher in sepsis survivors than in non-survivors [17].

During SARS-CoV-2 infection, the magnitude of the initial inflammatory response and a shift from Th1 toward a Th2-mediated immune response have also been associated with more severe disease [13].

#### Regulatory T cells (Tregs) (Fig. 2)

**Fig. 2:**
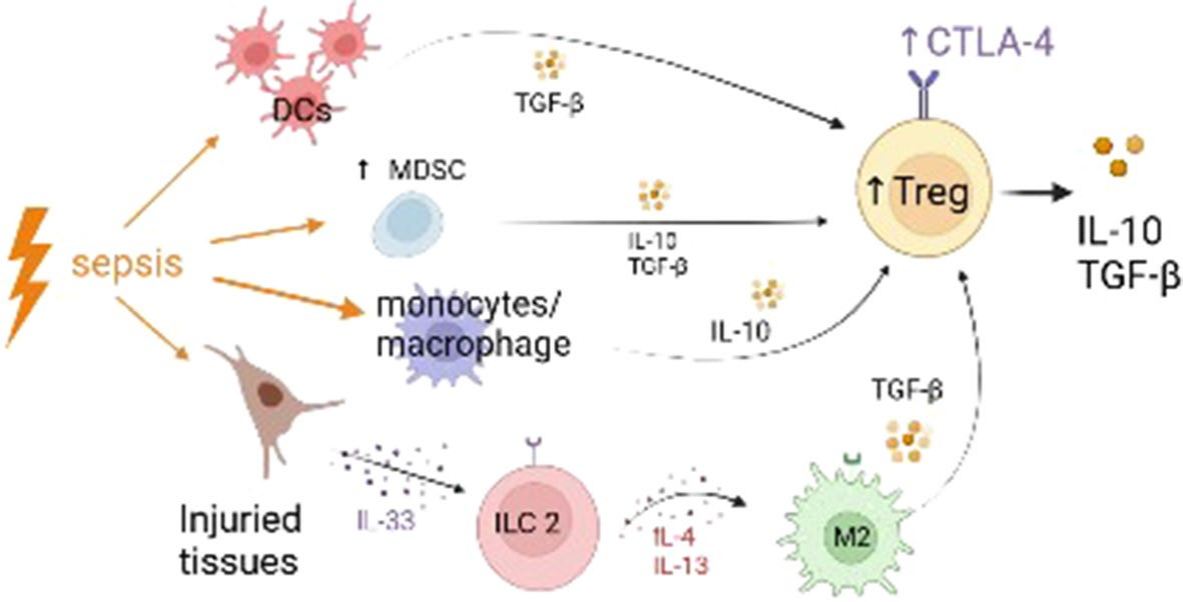
Mechanisms of regulatory T cells (Tregs) increase during sepsis. The increase of Tregs in sepsis is mediated by the production of TGF-β ± IL-10 by dendritic cells, monocytes/macrophages and MDSCs. Moreover, the injured tissues produce IL-33 which in turn activates ILC2 cells. Activated ILC2 cells release IL-4 and IL-13, cytokines responsible for a polarization of macrophages toward a M2 phenotype, which release TGF-β. DCs: dendritic cells; Treg: regulatory T cells; MDSC: myeloid-derived suppressor cells; TGF-β: transforming growth factor β; CTLA-4: cytotoxic T-lymphocyte-associated protein 4; IL-10: interleukin 10; IL-4: interleukin 4; IL-33: interleukin 33; ILC 2: type 2 innate lymphoid cells, M2: type 2 macrophage

Treg lymphocytes have a pivotal role in immune tolerance by controlling both the adaptive and innate immune responses. Treg lymphocytes encompass different subgroups of Tregs including natural and adaptive Tregs, as well as Type-1 T regulatory (Tr1) and T helper 3 (Th3) cells. Global Tregs identification is based on CD25, FOXP3 (forkhead box P3) and CD127 expression by CD4^+^ T cells (CD4^+^, CD25^+^, CD127^low^, FOX P3^high^ by flow cytometry). Suppressor function of Tregs is mainly mediated by the production of immunosuppressive cytokines—including IL (Interleukin)-10, and TGF-β (transforming growth factor β)—or through mechanisms of cell–cell contact via CTLA-4 (cytotoxic T-lymphocyte-associated protein 4) or glucocorticoid-induced TNF (tumor necrosis factor) receptor family-related protein (GITR) [18].

Increased Treg cells circulating in septic shock patients are responsible for lymphocyte anergy, with reduced lympho-proliferative response. In a cecal ligation and puncture model of sepsis in mice, Venet et al*.* showed that downregulation of Treg transcription factor Foxp3 was associated with a restoration of this response [19].

In an animal model of bacterial and viral pneumonia, Roquilly et al*.* have demonstrated that local dendritic cells have decreased antigen-presentation capacities and produce TGF-β after a primary infection, responsible for a massive recruitment of peripherally induced Treg and subsequent secondary immunosuppression with increased susceptibility to secondary infections [20]. The authors suggest that sepsis-induced immunosuppression may rather be due to a local immune reprogramming than an immune failure [20].

Another major contributor to the increase in Treg cells during sepsis is the interleukin IL-33. Nascimento et al*.* have shown that IL-33 released by injured tissues can activate type 2 innate lymphoid cells which induce the polarization of M2 macrophages, responsible for Treg cells expansion [21].

#### *T CD8* + *lymphocytes*

During sepsis, both naïve CD8 + T cells and memory CD8 T cells are impaired in quality and quantity, thus exposing patients to infections caused by previously encountered pathogens. Apoptosis of CD8 + cells is facilitated by cell expression of LFA-1 (lymphocytes function-associated antigen 1) [22]. Beyond apoptosis, CD8 + T cells undergo also phenotypic changes with reduced capacity of antigen-driven effector and proliferative functions [22]. Condotta et al. demonstrated that after polymicrobial sepsis in mice, CD8 + T cells express exhaustion markers such as PD-1 (programmed cell death protein 1), LAG-3 (lymphocyte-activation gene 3) and 2B4 (CD244), and produce less cytokines after antigen activation. These alterations predispose mice to chronic and secondary infections [23]

#### Innate T lymphocytes

Innate T cells, unconventional T lymphocytes with innate functions, are able to respond and proliferate rapidly (within hours) upon activation. They recognize non-peptide antigens presented by non-classical MHC (major histocompatibility complex) molecules, such as MHC I-like receptor (MR1), CD1d (a homologue of MHC I) and CD1a. Innate T cells are produced within the thymus and then reside in peripheral tissues [24]. Innate T cells include NKT (natural killer T) cells, MAIT (mucosal associated innate T) and γδT cells [24]. In humans, the most represented T innate cells are MAIT T cells, which have antigen specificity for riboflavin and folic acid metabolites presented by MR1. NK T cells recognize glycolipid antigens presented by CD1d [25]. MAIT T cells and NKT cells may both be activated by inflammatory cytokines, such as IL-12 and IL-18 [24]. γδ T cells can be activated by MHC 1 like molecules such as members of CD1 family, and by viral glycoproteins [26]. They are the most important epithelial lymphocytes in lung and intestine where they participate to gut and lung homeostasis, protect the mucosa of the intestine, and prevent the lungs from infections [27]. γδT cells can produce inflammatory cytokines, such as IL-17 and INF-γ (Interferon γ). Innate T cells play an important role in connecting innate and adaptive immune system and have been involved in the development of immunosuppression in critically ill patients [28]. Innate T cells activity is inhibited by Treg cells in sepsis [29]

MAIT cells have shown to be profoundly decreased in patients with bacterial infections, when compared to non-infected patients and healthy controls [30]. Moreover, persistent MAIT cell depletion has been associated with increased incidence of ICU-acquired infections [30].

γδT cells are also reduced in septic and non-septic ICU patients when compared to healthy subjects with no significant difference between septic and non-septic patients [30]. Tomasello et al*.* found a correlation between γδT cell reduction in sepsis and gut flora translocation, responsible for secondary infections [31]. Furthermore, γδT cells undergo phenotypic and functional changes during sepsis, such as decrease in CD69 expression and cytokine expression. The reduction of CD69 expression and INFγ production after antigen stimulation ex vivo has been associated with poor outcome [32].

On the opposite, there is not any quantitative change of NKT in infected or non-infected patients in ICU [30].

### Mechanisms of T lymphocytes dysregulation in critical diseases

1. ***Apoptosis***
  The main mechanism responsible for lymphopenia in severe inflammatory diseases, such as sepsis or SARS-CoV-2 infection, is increased lymphocyte apoptosis [14, 33, 34].
  In post-mortem studies, Hotchkiss et al*.* have first shown evidence of apoptosis in splenocytes of patients who died from sepsis [35]. They further demonstrated increased caspase-9 mediated apoptosis of B lymphocytes and T CD4 + cells in these patients, when compared to ICU non-septic patients [36]. The activation of caspase-9 in this study suggests a mitochondrial-mediated pathway involved in the initiation of apoptosis. Further experimental studies showing that overexpression of Bcl-2 (B-cell lymphoma 2) in T or B cells can prevent lymphocyte apoptosis in sepsis support these results [33, 37], as Bcl-2 may inhibit the mitochondrial but not the receptor-mediated apoptosis pathway [38]. Beyond sepsis, T cell apoptosis may also occur during hypoxia or ischemia–reperfusion [36]. Furthermore, T cells from septic patients have increased activity of caspase 8 and caspase 9 suggesting that apoptosis is mediated by both the intrinsic and the extrinsic pathway [39].
  Interestingly, Unsinger et al. have shown that sepsis-induced lymphocyte apoptosis does not require cognate antigen receptor interaction. Indeed, using TCR (T cell receptor) transgenic mice, they showed that classical activation-induced cell death (AICD) in which activation through the TCR with antigens or superantigens results in cell death, is not involved in sepsis-induced apoptosis. The authors suggest that lymphocyte apoptosis may be the result of a generalized, nonspecific loss of immune function [40].
  Lymphopenia in other critical diseases may also be mediated by AICD. Indeed, during SARS-CoV2 infection, Popescu et al*.* observed that CD4 + lymphopenia in COVID-19 patients was associated with worse outcome. They demonstrated in vitro that Spike 1 induces TNFα production in CD4 + specific cells. These TNFα + CD4 + cells have reduced proliferation and increased susceptibility to AICD. Treatment with infliximab or anti-TNF receptor 1 antibody was able to reverse AICD and increase CD4 + cells proliferation [41].
2. ***T-cell autophagy deficiency***
  Autophagy is another type of programmed cell death that is essential for cellular homeostasis. In an experimental study using T cell-specific autophagy gene Atg7 (Autophagy Related 7) knockout, Lin et al*.* demonstrated that T cell autophagy deficiency during sepsis contributed to sepsis-induced immunosuppression and mortality [42]. Oami et al*.* confirmed these results and showed that blocking autophagy using T cell-specific Atg5 (autophagy related 5) knockout mice accelerated T cell apoptosis, suggesting a protective role of T cell autophagy against apoptosis [43].
3. ***Anergy and exhaustion***
  Even after restoration of normal lymphocytes count, T cells functionality may still be impaired. This persistent condition is responsible for a prolonged immunosuppressive state [44–46]. Indeed, CD4 + and CD8 + T cells are less active and capable of producing cytokines such as IL- 6, INFγ, TNFα or IL-10 [45].
  Boomer et al*.* have observed an increased expression of exhaustion receptors, such as CTLA-4, TIM 3 (T-cell immunoglobulin and mucin containing protein-3) and LAG 3 on lymphocytes of septic patients, and a reduced production of INFγ by these cells, which was correlated to mortality and secondary infections [47].
  Moreover, CD4^+^ T lymphocytes express more PD-1 and PD-L1 (programmed death-ligand 1) in septic patients than trauma patients or healthy volunteers, with concomitant reduced lymphocyte proliferation and increased IL-10 secretion with a greater incidence of secondary infections and increased mortality [48].
  Another mechanism of immune impairment observed after major trauma, burns or sepsis is T cell anergy [29]. Anergy is characterized by the inability of lymphocytes to recognize the cognate antigen, to activate, proliferate and produce cytokines. In a recent study, Guinault et al. have studied CD8^+^ T cell exhaustion markers (2B4, PD-1, and CD160 markers) in septic patients and identified 3 clusters of patients according to their expression of exhaustion markers. They found that 2B4^hi^ PD1 × CD160 CD8^+^ T cells patterns were strongly associated with mortality, with the 2B4^hi^PD1^hi^CD160^low^ pattern characterized by a strong reduction in cytokine secretion [49].
4. ***Lymphocyte metabolism***
  In the last decade, there has been growing evidence that alterations in lymphocyte metabolism may contribute to lymphocyte dysfunction. Using transcriptional and metabolic profiling of septic patients, Cheng et al*.* have reported a shift from oxidative phosphorylation to aerobic glycolysis in activated lymphocytes. A metabolic defect of both glycolysis and oxidative phosphorylation may contribute to secondary immunoparalysis. Moreover, they found that immunologic recovery correlated with metabolic recovery [50].

In a recent study, Reizine et al*.* have shown, in a cecal ligation and puncture model in mice, that T cells had decreased maximal mitochondrial oxygen consumption rate and reduced adenosine triphosphate production, with subsequent T cell apoptosis, exhaustion and Treg increase.

They showed that arginine deficiency plays a crucial role in these metabolic changes, as citrulline (a precursor of arginine) supplementation was able to reduce T cell apoptosis, increase T cell proliferation and reduce the accumulation of Treg cells. These effects were associated with a restoration of T cell metabolism and a more efficient T cell function, with improvement of acute lung injury during secondary bacterial pneumonia [51].

Recently, Karagiannis et al. have highlighted the relationship between impaired metabolic adaptation to acute infections and immune response. They focused their study on ketogenesis (and production of ketonic bodies, including β-hydroxybutyrate (BHB)) resulting from fasting and anorexia as an adaptive mechanism to pulmonary infections. They found that the production of BHB was impaired in patients with SARS-CoV-2-induced acute respiratory distress syndrome. They demonstrated that BHB is crucial as an alternative carbon source that can preserve CD4 + cell response and INFγ production in acute pulmonary infections [52].

### Innate immune system: other actors and their impact on T cell regulation

1. ***Monocytes, macrophages, and dendritic cells***
  The phenotype and functions of monocytes, macrophages and dendritic cells are also altered during sepsis, with decreased effector functions, decrease in antigen-presentation capacities, as assessed by reduced HLA-DR (Human Leukocyte Antigen-DR isotype) expression, increased production of anti-inflammatory mediators (mainly IL-10) and increased expression of immune checkpoint inhibitors. These changes result in altered T cells activity and proliferation and are associated with worse outcome [53, 54].
2. ***Myeloid-derived suppressor cells (MDSCs)***
  MDSCs have initially been described as responsible for T cell dysfunction and tolerance in cancer and chronic infections but had successively emerged as a major player in sepsis-induced immunosuppression. MDSCs are immature myeloid cells comprising progenitors or precursors of dendritic cells, monocytes, and neutrophils. Delano et al*.* have first identified this population of GR-1^+^CD11b^+^ myeloid cells in spleen, bone marrow and lymph nodes of septic mice. This population, composed of immature myeloid cells, expresses IL-10, TGFβ and TNFα. They are responsible for Th2 polarization of CD4 + cell response and for the inhibition of CD8 + cell proliferation and antigen-specific IFNγ production by CD8^+^ T lymphocytes [15]. They also prevent NK cells from producing INFγ [55]. Uhel et al*.* have shown that increased number of MDSCs in blood was strongly associated with the occurrence of nosocomial infections in septic patients [56]. Similarly, an increased number of MDSCs in patients with SARS-CoV-2-related acute respiratory distress syndrome has been associated with lymphopenia and a decrease in CD8 + effector memory cells [57].

### Potential therapeutic approaches

In the last decade, efforts have concentrated on reducing sepsis-induced lymphocyte cell death and increasing T cell functionality using immunostimulatory strategies, such as INFγ, IL-7, or checkpoints inhibitors [58]. Combining treatments that act both on lymphocyte apoptosis and exhaustion may also have a synergic effect [59]. Although preliminary results of pilot studies are encouraging, they will require further confirmation in larger clinical trials.

IL-7 is a cytokine that is crucial for T cell survival, proliferation, and effector functions. It has therefore been proposed as an adjuvant therapy in septic patients [59, 60]. In a prospective randomized trial in 27 patients with sepsis and severe lymphopenia, François et al*.* found that recombinant IL-7 was able to increase CD4 and CD8 T cell number and was well tolerated [61].

Immune checkpoint inhibitors, such as anti-PD1, anti-PDL1, anti-CTLA4, anti-TIM3 and anti-LAG3 antibodies have also been considered as promising therapies in patients with sepsis. Experimental studies in mice and ex vivo studies in humans have highlighted the efficacy of these treatments in improving T cell function [62–67].

In a case report of post-septic immunosuppression after trauma, a patient with refractory mucormycosis was successfully treated by an anti-PD-1 monoclonal antibody and INFγ [68].

In a phase 1 randomized study, Hotchkiss et al*.* found that anti-PD-L1 inhibitors were well tolerated and were able to partially restore immune status over 28 days in patients with sepsis-induced immunosuppression [69].

IFNγ is produced by Th1 cells, activates monocytes and increases their antigen-presentation capacity. In severe COVID-19 patients, some authors have suggested that IFNγ therapy was able to decrease SARS-CoV-2 load and improve outcome [70]. However, large, randomized studies testing the efficacy of anti-PDL1 inhibitors and IFNγ therapy are still warranted to prove their efficacy in critically ill patients. Close monitoring of immune and inflammatory responses before and after immunoadjuvant therapies are necessary to target patients with lymphopenia and avoid drug-induced overwhelming inflammatory responses.

## Conclusion

Innate and adaptive immune responses are crucial in inflammatory critical illnesses. T cell dysregulation has a pivotal role in the pathogenesis of post-aggressive immunosuppression, contributing to secondary infections and increased morbidity and mortality. Quantitative and qualitative kinetic monitoring of T lymphocytes in ICU patients may help to identify those who may benefit from new immunomodulatory therapeutic strategies [71].

## Data Availability

Not applicable.

## References

[1] Chousterman BG, Swirski FK, Weber GF (2017). Cytokine storm and sepsis disease pathogenesis. Semin Immunopathol.

[2] Munford RS, Pugin J (2001). Critical care perspective normal responses to injury prevent systemic inflammation and can be immunosuppressive. Am J Respir Crit Care Med.

[3] Roquilly A, Villadangos JA (2015). The role of dendritic cell alterations in susceptibility to hospital-acquired infections during critical-illness related immunosuppression. Mol Immunol.

[4] Adrie C, Lugosi M, Sonneville R (2017). Persistent lymphopenia is a risk factor for ICU-acquired infections and for death in ICU patients with sustained hypotension at admission. Ann Intensive Care.

[5] Drewry A, Samra N, Skrupky L (2014). Persistent lymphopenia after diagnosis of sepsis predicts mortality. Shock.

[6] Ferguson NR, Galley HF, Webster NR (1999). T helper cell subset ratios in patients with severe sepsis. Intensive Care Med.

[7] Otto GP, Sossdorf M, Claus RA (2011). The late phase of sepsis is characterized by an increased microbiological burden and death rate. Crit Care.

[8] Walton AH, Muenzer JT, Rasche D (2014). Reactivation of multiple viruses in patients with sepsis. PLoS ONE.

[9] Van Vught LAV, Klouwenberg PMCK, Spitoni C (2016). Incidence, risk factors, and attributable mortality of secondary infections in the intensive care unit after admission for sepsis. J Am Med Assoc.

[10] Choi YJ, Kim SB, Kim JH (2017). Impaired polyfunctionality of CD8+ T cells in severe sepsis patients with human cytomegalovirus reactivation. Exp Mol Med.

[11] Hohlstein P, Gussen H, Bartneck M (2019). Prognostic relevance of altered lymphocyte subpopulations in critical illness and sepsis. J Clin Med.

[12] Monserrat J, de Pablo R, Reyes E (2009). Clinical relevance of the severe abnormalities of the T cell compartment in septic shock patients. Crit Care.

[13] Lucas C, Wong P, Klein J (2020). Longitudinal analyses reveal immunological misfiring in severe COVID-19. Nature.

[14] Zhang S, Asquith B, Szydlo R (2021). Peripheral T cell lymphopenia in COVID-19: potential mechanisms and impact. Immunother Adv.

[15] Delano MJ, Scumpia PO, Weinstein JS (2007). MyD88-dependent expansion of an immature GR-1 +CD11b+ population induces T cell suppression and Th2 polarization in sepsis. J Exp Med.

[16] Watanabe H, Numata K, Ito T (2004). Innate immune response in Th1- and Th2-dominant mouse strains. Shock.

[17] de Pablo R, Monserrat J, Prieto A, Alvarez-Mon M (2014). Role of circulating lymphocytes in patients with sepsis. Biomed Res Int.

[18] Jiang LN, Yao YM, Sheng ZY (2012). The role of regulatory T cells in the pathogenesis of sepsis and its clinical implication. J Interferon Cytokine Res.

[19] Venet F, Chung CS, Kherouf H (2009). Increased circulating regulatory T cells (CD4+CD25 +CD127-) contribute to lymphocyte anergy in septic shock patients. Intensive Care Med.

[20] Roquilly A, McWilliam HEG, Jacqueline C (2017). Local modulation of antigen-presenting cell development after resolution of pneumonia induces long-term susceptibility to secondary infections. Immunity.

[21] Nascimento DC, Melo PH, Piñeros AR (2017). IL-33 contributes to sepsis-induced long-Term immunosuppression by expanding the regulatory T cell population. Nat Commun.

[22] Danahy DB, Strother RK, Badovinac VP, Griffith TS (2016). Clinical and experimental sepsis impairs CD8 T-Cell-mediated immunity. Crit Rev Immunol.

[23] Condotta SA, Khan SH, Rai D (2015). Polymicrobial sepsis increases susceptibility to chronic viral infection and exacerbates CD8 + T Cell Exhaustion. J Immunol.

[24] Kim EY, Oldham WM (2019). Innate T cells in the intensive care unit. Mol Immunol.

[25] Cohen NR, Garg S, Brenner MB (2009). Chapter 1 Antigen Presentation by CD1. Lipids, T Cells, and NKT Cells in Microbial Immunity. Adv Immunol.

[26] Kalyan S, Kabelitz D (2013). Defining the nature of human γδ T cells: a biographical sketch of the highly empathetic. Cell Mol Immunol.

[27] Brady J, Horie S, Laffey JG (2020). Role of the adaptive immune response in sepsis. Intensive Care Med Exp.

[28] Szabo PA, Anantha R, v., Shaler CR,  (2015). CD1d- and MR1-restricted T Cells in Sepsis. Front Immunol.

[29] Hotchkiss RS, Monneret G, Payen D (2013). Sepsis-induced immunosuppression: From cellular dysfunctions to immunotherapy. Nat Rev Immunol.

[30] Grimaldi D, le Bouhris L, Sauneuf B (2014). Specific MAIT cell behaviour among innate-like T lymphocytes in critically ill patients with severe infections. Intensive Care Med.

[31] Tomasello E, Bedoui S (2013). Intestinal innate immune cells in gut homeostasis and immunosurveillance. Immunol Cell Biol.

[32] Liao XL, Feng T, Zhang JQ (2017). Phenotypic changes and impaired function of peripheral γδ T cells in patients with sepsis. Shock.

[33] Hotchkiss RS, Tinsley KW, Swanson PE (1999). Prevention of lymphocyte cell death in sepsis improves survival in mice. Proc Natl Acad Sci USA.

[34] le Tulzo Y, line Pangault C, Gacouin A,  (2002). Early circulating lymphocyte apoptosis in human septic shock is associated with poor outcome. Shock.

[35] Hotchkiss RS, Swanson PE, Freeman BD (1999). Apoptotic cell death in patients with sepsis, shock, and multiple organ dysfunction. Crit Care Med.

[36] Hotchkiss RS, Tinsley KW, Swanson PE (2001). Sepsis-induced apoptosis causes progressive profound depletion of B and CD4 + T Lymphocytes in Humans. J Immunol.

[37] Karl Kimberly M, Zollner IE, Buchman TG, Chang KC, SJ,  (1999). Sepsis in decreases apoptosis and improves survival overexpression of bcl-2 in transgenic mice. Research.

[38] Roy S, Nicholson DW (2000) Commentary cross-talk in cell death signaling 11034597

[39] Hotchkiss RS, Osmon SB, Chang KC (2005). Accelerated lymphocyte death in sepsis occurs by both the death receptor and mitochondrial pathways. J Immunol.

[40] Unsinger J, Herndon JM, Davis CG (2006). The ROLE of TCR engagement and activation-induced cell death in sepsis-induced T Cell Apoptosis. J Immunol.

[41] Popescu I, Snyder ME, Iasella CJ (2022). CD41 T-Cell Dysfunction in Severe COVID-19 disease is tumor necrosis factor-a/tumor necrosis factor receptor 1–dependent. Am J Respir Crit Care Med.

[42] Lin CW, Lo S, Hsu C (2014). T-cell autophagy deficiency increases mortality and suppresses immune responses after sepsis. PLoS ONE.

[43] Oami T, Watanabe E, Hatano M (2017). Suppression of T Cell autophagy results in decreased viability and function of T Cells through accelerated apoptosis in a murine sepsis model. Crit Care Med.

[44] Jensen IJ, Sjaastad F, v, Griffith TS, Badovinac VP,  (2018). Sepsis-Induced T cell immunoparalysis: the ins and outs of impaired T cell immunity. J Immunol.

[45] Boomer JS, To K, Chang KC (2022). Immunosuppression in Patients Who Die of Sepsis and Multiple Organ Failure. Gut.

[46] Ward PA (2012). New approaches to the study of sepsis. EMBO Mol Med.

[47] Boomer JS, Shuherk-Shaffer J, Hotchkiss RS, Green JM (2012). A prospective analysis of lymphocyte phenotype and function over the course of acute sepsis. Crit Care.

[48] Guignant C, Lepape A, Huang X (2011). Programmed death-1 levels correlate with increased mortality, nosocomial infection and immune dysfunctions in septic shock patients. Crit Care.

[49] Guinault N-T-L, Silva S (2021). Expression of Exhaustion Markers on CD8+ T-Cell Patterns Predict Outcomes in Septic Patients Admitted to the ICU. Crit Care Med.

[50] Cheng SC, Scicluna BP, Arts RJW (2016). Broad defects in the energy metabolism of leukocytes underlie immunoparalysis in sepsis. Nat Immunol.

[51] Reizine F, Grégoire M, Lesouhaitier M (2022). Beneficial effects of citrulline enteral administration on sepsis-induced T cell mitochondrial dysfunction. PNAS.

[52] Karagiannis F, Peukert K, Surace L (2022). Impaired ketogenesis ties metabolism to T cell dysfunction in COVID-19. Nature.

[53] Venet F, Monneret G (2018). Advances in the understanding and treatment of sepsis-induced immunosuppression. Nat Rev Nephrol.

[54] Monneret G, Finck ME, Venet F (2004). The anti-inflammatory response dominates after septic shock: Association of low monocyte HLA-DR expression and high interleukin-10 concentration. Immunol Lett.

[55] Pastille E, Didovic S, Brauckmann D (2011). Modulation of dendritic cell differentiation in the bone marrow mediates sustained immunosuppression after polymicrobial sepsis. J Immunol.

[56] Uhel F, Azzaoui I, Grégoire M (2017). Early expansion of circulating granulocytic myeloid-derived suppressor cells predicts development of nosocomial infections in patients with sepsis. Am J Respir Crit Care Med.

[57] Reizine F, Lesouhaitier M, Gregoire M (2021). SARS-CoV-2-Induced ARDS Associates with MDSC expansion, lymphocyte dysfunction, and arginine shortage. J Clin Immunol.

[58] Vincent JL, Grimaldi D (2018). Novel Interventions: What’s New and the Future. Crit Care Clin.

[59] Martin MD, Badovinac VP, Griffith TS (2020). CD4 T Cell Responses and the Sepsis-Induced Immunoparalysis State. Front Immunol.

[60] Unsinger J, McGlynn M, Kasten KR (2010). IL-7 Promotes T cell viability, trafficking, and functionality and improves survival in sepsis. J Immunol.

[61] Francois B, Jeannet R, Daix T (2018). Interleukin-7 restores lymphocytes in septic shock: the IRIS-7 randomized clinical trial. JCI Insight.

[62] Patera AC, Drewry AM, Chang K (2016). Frontline Science: Defects in immune function in patients with sepsis are associated with PD-1 or PD-L1 expression and can be restored by antibodies targeting PD-1 or PD-L1. J Leukoc Biol.

[63] Shindo Y, McDonough JS, Chang KC (2017). Anti-PD-L1 peptide improves survival in sepsis. J Surg Res.

[64] Chang K, Svabek C, Vazquez-Guillamet C (2014). Targeting the programmed cell death 1: programmed cell death ligand 1 pathway reverses T cell exhaustion in patients with sepsis. Cell.

[65] Chang KC, Burnham CA, Compton SM (2013). Blockade of the negative co-stimulatory molecules PD-1 and CTLA-4 improves survival in primary and secondary fungal sepsis. Crit Care.

[66] Brahmamdam P, Inoue S, Unsinger J (2010). Delayed administration of anti-PD-1 antibody reverses immune dysfunction and improves survival during sepsis. J Leukoc Biol.

[67] Zhang Y, Zhou Y, Lou J (2010). PD-L1 blockade improves survival in experimental sepsis by inhibiting lymphocyte apoptosis and reversing monocyte dysfunction. Crit Care.

[68] Grimaldi D, Pradier O, Hotchkiss RS, Vincent JL (2017). Nivolumab plus interferon-γ in the treatment of intractable mucormycosis. Lancet Infect Dis.

[69] Hotchkiss RS, Colston E, Yende S (2019). Immune checkpoint inhibition in sepsis: a phase 1b randomized, placebo-controlled, single ascending dose study of antiprogrammed cell death-ligand 1 Antibody (BMS-936559). Crit Care Med.

[70] van Laarhoven A, Kurver L, Overheul GJ (2021). Interferon gamma immunotherapy in five critically ill COVID-19 patients with impaired cellular immunity: A case series. Med.

[71] Rol ML, Venet F, Rimmele T (2017). The REAnimation Low Immune Status Markers (REALISM) project: A protocol for broad characterisation and follow-up of injury-induced immunosuppression in intensive care unit (ICU) critically ill patients. BMJ Open.

